# Tobacco advertising, cross-over effects, and US adolescent progression from never to current tobacco use

**DOI:** 10.1186/s12889-025-26064-y

**Published:** 2026-01-19

**Authors:** Dennis R. Trinidad, John P. Pierce, Brian Dang, David R. Strong, Matthew D. Stone, Sara B. McMenamin, Thet Nwe Myo Khin, Karen Messer

**Affiliations:** 1 Herbert Wertheim School of Public Health and Human Longevity Science, University of California San Diego, La Jolla, California 92093 USA; 2 Moores Cancer Center, University of California San Diego, 3855 Health Sciences Drive, La Jolla, San Diego, California 92093 USA

**Keywords:** Tobacco, Advertising, Receptivity, Adolescents, Cigarettes, Electronic cigarettes

## Abstract

**Background and objectives:**

Adolescent receptivity to tobacco advertising has been linked to increased tobacco initiation in longitudinal studies. However, not all ever users progress to daily use. We examined whether receptivity to tobacco advertising among adolescents was associated with product-specific use, including daily use, as an adult, and whether receptivity to one product had a cross-over effect and predicted use of a different product at follow-up.

**Methods:**

In the US Population Assessment of Tobacco and Health (PATH) Study, 74.6% of adolescent never-tobacco-users at baseline (2013-14) reported receptivity to tobacco advertising (for cigarettes, e-cigarettes, cigars, smokeless tobacco) and were surveyed on current tobacco use (every day, some days in past 30 days) an median of 7 years later (*n* = 7506). Multivariable logistic regression analyses included 8 common covariates.

**Results:**

At follow-up, 20.1% were current tobacco users (15.4% e-cigarettes) and 8.4% were daily users (6.5% e-cigarettes). Receptivity to any advertising at baseline was associated with current use (AOR = 1.46, 95%CI: 1.29,1.66) as well as daily use (AOR = 1.41, 95%CI: 1.16,1.72). Product-specific advertising was associated with current use of each product at follow-up and there was a cross-over effect with receptivity to product advertising associated with current use of a different product. This cross-over effect on progression to daily use was only seen for e-cigarettes (e-cigarette receptivity: AOR = 1.48, 95%CI: 1.19,1.84; cross-over receptivity: AOR = 1.55, 95%CI: 1.16,2.06). The usual e-cigarette device for current vapers at follow-up included disposables (37.6%), refillable tanks (27.9%) and cartridges (26.4%). Fruit/candy and menthol flavors were used most. JUUL was the most common e-cigarette brand and 29% of JUUL users recently vaped fruit/candy flavors.

**Conclusions:**

Among adolescents who were receptive to tobacco advertising but had never used tobacco at baseline, there was significant progression to current and daily tobacco use 6.6 years later. The cross-over advertising effect went beyond the particular advertised product and effectively promoted daily nicotine use, particularly among 12-14-year-old adolescents at baseline, with progression to e-cigarettes. Population increases in adolescent progression to daily nicotine use is a public health harm that needs public health action to counteract the effectiveness of e-cigarette marketing both in schools and at the community level.

**Supplementary Information:**

The online version contains supplementary material available at 10.1186/s12889-025-26064-y.

## Introduction

 Innovative tobacco advertising campaigns on mainstream media throughout the 20th century were associated with increased tobacco initiation among targeted youth [1–4]. Longitudinal studies of US adolescents in the 1990s documented that receptivity to cigarette advertisements predicted which never-smoking adolescents would first experiment [5] and then progress [6] towards dependence on cigarette smoking [7]. Tobacco advertisements appear to be particularly appealing at the age when young adolescents are developing their own self-concept [8]. With the research proving that marketing was effective in encouraging use and internal tobacco industry documents clearly outlining that industry marketing targeted adolescents [9–12], cigarette marketing restrictions were included in the 1998 Master Settlement Agreement (MSA) reached between state attorneys general and the tobacco industry [13]. The implementation of this MSA and the conduct of successful public health campaigns at both the US state [14, 15] and federal levels [16, 17] were associated with the start of a long decline in cigarette smoking in adolescents and young adults [18–20] and, in 2012, the US Surgeon General concluded that tobacco advertising was causally associated with both initiation and progression to cigarette smoking among young people [21]. 

However, in the last decade, there have been major changes particularly to the channels used by tobacco marketers and their message sources, although the overall goal has remained the same [22] – to build receptivity and engagement [23, 24] in the target audience. The channels changed because the mainstream media (e.g., broadcasters and newspapers) have been struggling to reach and build relationships with those under age 35 years, who increasingly get their news through digital social media such as facebook, Youtube, WhatsApp (each of which had over one billion users in 2019) [25, 26]. The first documented successful social media marketing of a consumer product occurred on facebook in 2012 and demonstrated the value of user interactivity that social media offered to marketing [27]. The last decade has seen the rise of social media influencers as a major message source for tobacco advertising [28], each of who had an established large following before being cultivated as product brand ambassadors. These have taken over from the role movie stars had in advertising on mainstream media [29]. 

Early research considered peers as the primary influence on smoking initiation where nonsmokers started smoking to obtain social acceptance from their peers who already smoked [30]. However, friendship groups change during adolescence [31–34] and receptivity to advertising has been shown to precede the seeking of a friendship group that includes cigarette smokers [35]. Thus, peer influence appears to be a mediator between receptivity to marketing and tobacco use behavior. Prior to conducting a mediational analysis, we need to establish that receptivity is associated with later adult tobacco use. The prevalence of tobacco use in US youth varies with age, sex, race/ethnicity, and education [21], and is more likely among those with symptoms of potential mental health problems [36]. Other variables shown to affect tobacco use among youth include exposure to a tobacco user at home [37] and a lack of a smoke-free home [38]. There are similar use patterns with e-cigarettes [39]. 

Public health concerns about unrestricted e-cigarette marketing, particularly on social media, were already being expressed in 2010 [23, 40]. By 2013/14, adolescent receptivity to e-cigarette marketing had surpassed receptivity to cigarette marketing [41]. Receptivity to advertising for different tobacco products has been demonstrated to be associated with use of that product over the following couple of years [42–47]. However, not all ever users progress to nicotine dependence [7], which is characterized by craving, tolerance (including to daily use) and loss of control [48]. Through 2016, new daily tobacco use in the 14–24-year-old population occurred primarily with cigarette smoking [49] and it was in that year that the Food and Drug Administration (FDA) was given the authority to regulate e-cigarettes [50]. The following year (2017), JUUL Labs, Inc. led the surge in e-cigarette sales with their trend-setting social media marketing of an innovative, high nicotine, flavored e-cigarette [51]. Other US e-cigarette manufacturers quickly increased the nicotine content of their e-cigarettes and also added flavors [52]. Between November 2016 and August 2019, e-cigarette unit sales increased by nearly 300% [53] and, as cigarette smoking continued to decline [54] e-cigarettes became the predominant tobacco product used by US youth [55, 56]. 

In this study, we extend the previous research by following 12–17-year-old participants from Wave 1 (W1) of the Population Assessment of Tobacco and Health (PATH) Study through adulthood at W6. We report whether receptivity as a young adolescent was associated with use of that product, including daily use, as an adult. As all products are nicotine delivery systems, we hypothesize that receptivity to one product will cross-over and predict use of a different product at follow-up. In particular, as during the follow-up period, young adult cigarette smoking declined [57] and e-cigarette vaping increased substantially [58], we hypothesize that receptivity to a tobacco product other than e-cigarettes at Wave 1 would be associated with daily e-cigarette vaping at wave 6. Finally, we report the most popular e-cigarette brands that were vaped by these young adults at Wave 6.

## Methods

### Data source

The PATH Study is a large, nationally representative, longitudinal study on tobacco product use among the US population aged 12 + years [59]. Following an in-person household screener survey, up to two 12–17-year-olds were scheduled for interview along with a stratified sample of adults. Audio-computer-assisted self-interviews in English or Spanish collected self-report information on tobacco-use patterns and associated health behaviors [60]. At W1 (2013-14) we identified 10,246 12–17-year-old respondents who had never used any tobacco products. We required that they had completed their latest follow-up PATH survey at either W6 (2021, *n* = 5703, median follow-up = 85 months) or, if not, then W5 (2019, an additional *n* = 1803, median follow-up = 60 months). For the whole sample, the median length of follow up was 84 months (range: 52 to 98). Non-completion of follow-up (W5 or W6) was 25.4% (95% CI: 24.3%, 26.5%) with lower rates for the 12–14 age-cohort than the 15–17 age cohort, for race/ethnic minorities than non-Hispanic Whites, and for those who were receptive to tobacco product advertising than those who were non-receptive (eTable 1). For survey respondents under 18 years old, adolescents assented to each interview and parental consent was obtained in a linked parental survey. Adults provided informed consent at each interview. The study was conducted by Westat and approved by the Westat Institutional Review Board.

### Study measures

#### Identifying never-tobacco-users at W1

All 12–17-year-old respondents were asked if they had ever used a cigarette, even 1 or 2 puffs. Respondents were also shown separate pictures for e-cigarettes, cigars, smokeless tobacco, pipe, hookah, dissolvable products, bidis and/or kreteks and asked whether they had ever used the product, even 1 or 2 times (“yes” or “no” for each product). To be considered a never-tobacco-user, the respondent must have indicated never using all the products listed.

#### Tobacco use at follow-up

At each survey wave, ever use was assessed for each tobacco product, with “Have you ever [smoked/vaped/used], even 1 or 2 times? Adolescents at follow-up were asked, “In the past 30 days, how many days did you use this product?” Those who reported using a product for at least 1 day were classified as current users. As in past research [61, 62], we used an expansive definition of daily use for study participants that were still aged 12–17 years at follow-up (W5 = 1,559, W6 = 4), considering > 25 of the past 30 days to be daily use. The vast majority of our sample had aged into the adult survey (18 + years) by W5 or W6 and were asked “Have you ever [smoked/vape/used], even 1 or 2 times?” and “Do you now [smoke/vape/use] every day, some days or not at all?” Those adults at follow-up who reported smoking/vaping/using every day or some days were classified as current users. Those who reported smoking/vaping/using every day were daily users. Those who currently vaped e-cigarettes were asked their device type (disposable device, replacement pods or cartridges, refillable tank, a mod system, other), brand, type of device and flavors used in the past 30 days (tobacco, menthol, mint, clove/spice, fruit, chocolate, an alcoholic drink flavor, non-alcoholic drink flavor, candy/dessert).

#### Receptivity to tobacco product advertising

At W1, each respondent was shown 20 randomly selected ads from a census of ads in that year (5 each for cigarettes, e-cigarettes, cigars and smokeless tobacco) from a near-census collection (*n* = 959) of print, direct mail, and television ads used in the US in the previous year [42]. For each ad, respondents were asked if they had seen it in the past year (aided recall) and whether they liked, disliked, or were neutral to the ad. Variables for whether youth respondents were able to recall the ad (“have you seen this advertisement in the past 12 months prior to this study,” response options were “yes” or “no”) and their liking the advertisement (“how much do you like this advertisement,” response options were “like this ad,” “no opinion,” and “dislike this ad”) were constructed separately. Advertisement receptivity was ascertained as follows: any respondent indicating they like the advertisement was encoded as high receptivity, regardless of if they recalled the advertisement; any respondent who could recall the ad, but were neutral to or disliked the ad were treated as low receptivity; and no recall and were neutral or disliked the ad were classified as nonreceptive. Any recall or high or low receptivity was coded as being receptive to the product’s advertising. This measure of receptivity has been shown to have predictive validity for both susceptibility and trial of each product in prior studies [41, 42]. 

#### Other variables

The PATH Study imputes missing data on age, sex, and race/ethnicity from other study data and we use the derived variables in the PATH Study Data User Guide [60]. Other covariates included parental education, household tobacco use, smokefree home, internalizing and externalizing mental health symptoms (see eTable 2 for measurement details).

### Statistical analysis

We computed variances using the recommended balanced repeated replication method with Fay adjustment = 0.3 [63]. We calculated weighted percentages and Wald-type confidence intervals (CIs) in the log-odds scale and transformed them to a probability scale. For each product with receptivity data, we used logistic regression models to test whether W1 receptivity (compared with no receptivity) was associated with progression to ever use, current use or daily use at follow-up. As adult males (4.2%) were much more likely to use smokeless products than adult females (0.2%) [64], we conducted the smokeless models for males only. All logistic regressions included W1 covariates for age, sex, race/ethnicity, parental education, living with a tobacco user, and household tobacco rules. There was no effect of time to follow-up in any models. Because we note the effects of age cohort at W1 on progression to current or daily tobacco use at follow-up, we did not include the respondent’s age at follow-up in the logistic regression model. Odds ratios, 95%CIs, and p-values were reported from the weighted adjusted model. All analyses were conducted in R, version 4.3.2, using the ‘survey’ package, version 4.0.

## Results

### Receptivity to tobacco advertising in two age cohorts

**Fig. 1:**
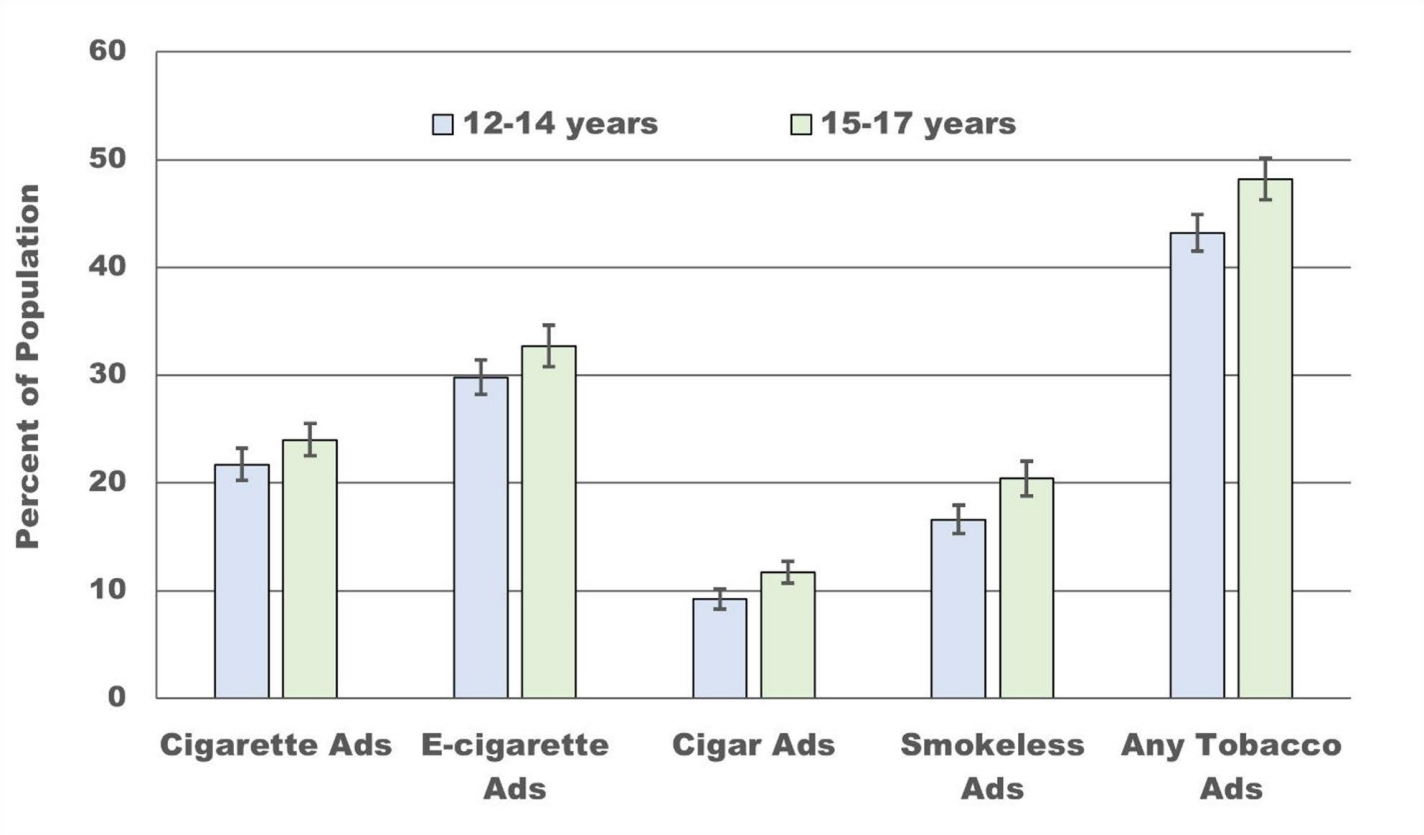
Adolescent never-tobacco-users aided recall/liking of at least 1 of the 5 randomly selected tobacco ads for each of cigarettes, e-cigarettes, cigars and smokeless tobacco products in wave 1 of US PATH Study (2013-14)

At baseline (W1), receptivity to tobacco product advertising among US adolescent never-tobacco-users was highest for e-cigarette ads (31.0%, 95%CI: 29.8%,32.2%) followed by cigarette ads (22.7%, 95%CI: 21.6%,23.8%), smokeless tobacco ads (18.2%, 95%CI: 17.1%,19.3%) and cigars ads (10.3%, 95%CI: 9.6%,11.0%). Almost half (45.3%, 95%CI: 43.9%,46.7%) were receptive to at least one tobacco product’s advertisement (Fig. 1, eTable 3). Receptivity to any tobacco advertisement was higher among 15–17-year-olds (48.2%, 95%CI: 46.2%,50.1%) than 12–14-year-olds (43.2%, 95%CI: 41.4%,44.9%) although there was no difference between these ages in receptivity to either cigarettes (21.7% vs. 24.0%) or e-cigarettes (29.8% vs. 32.7%).

### Progression to using a tobacco product at follow-up by age

**Fig. 2:**
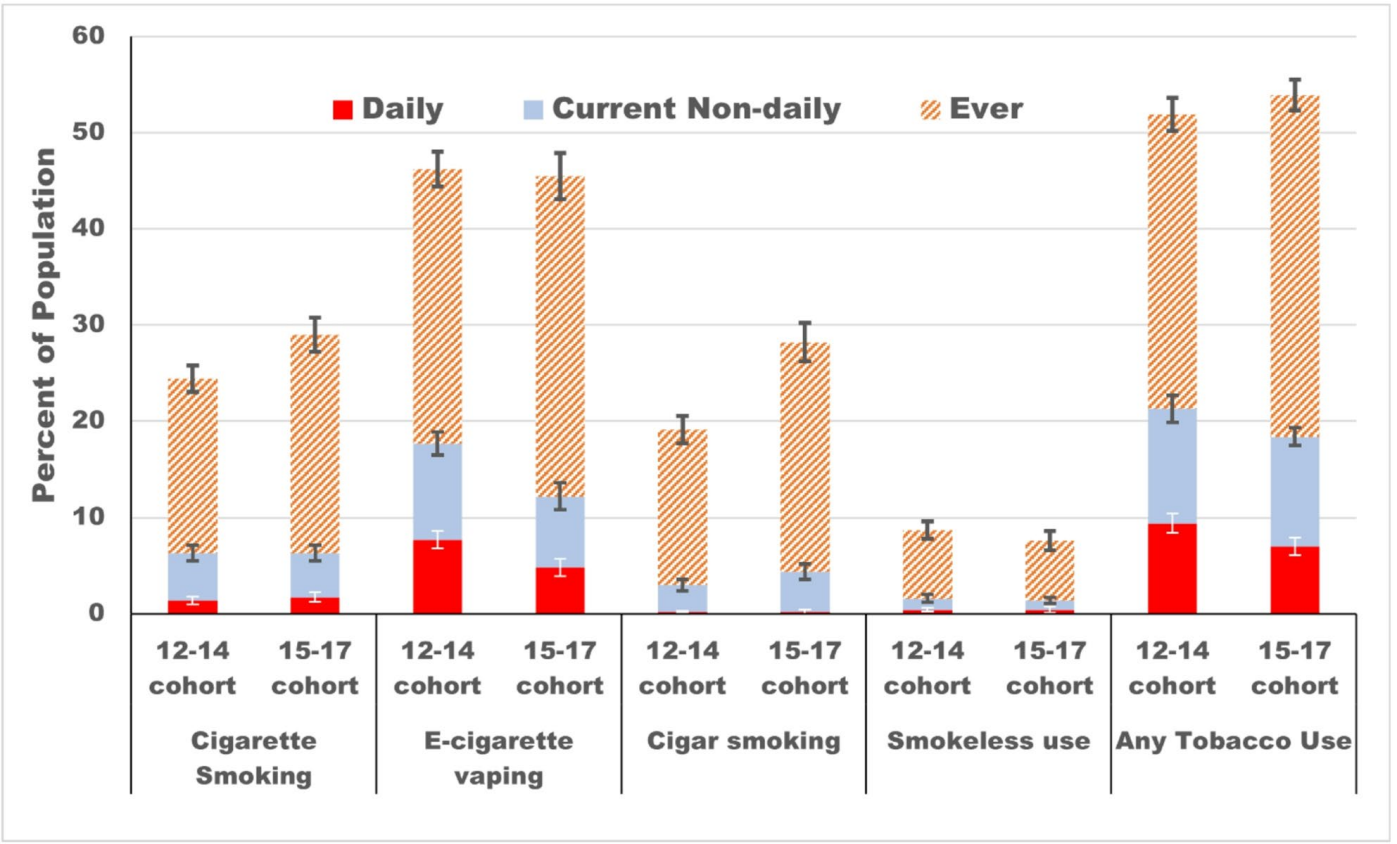
Progression to different levels of tobacco use over 7 years of follow-up [median follow-up = 84 months, range 52–98 months] among two age cohorts of adolescent never-tobacco-users identified at wave 1 of US PATH Study

After a median of 7 years, half of both the 12–14 (51.9%, 95%CI: 50.1,53.6) and 15–17 (53.9%, 95%CI: 51.7%,56.2%) age cohorts reported being ever tobacco users (Fig. 2, eTable 4). However, the 12–14 age cohort appeared to have a higher prevalence of both daily as well as current tobacco use. In the 12–14 age cohort, > 80% of both daily (7.7/9.4) and current use (17.7/21.3) was e-cigarette vaping, compared to < 70% for the 15–17 cohort (daily = 4.8/7.0, current = 12.2/18.4).

Separate models for progression to current and daily tobacco use at follow-up, each adjusted for all study covariates, are presented in Table 1. After this adjustment, the 12–14 age cohort had higher odds than the 15–17 age cohort of being a current user at follow-up (AOR = 1.22, 95%CI: 1.07,1.40), but much higher odds of being daily tobacco users (AOR = 1.41, 95%CI: 1.17,1.70). Both internalizing and externalizing mental health symptoms were associated with much higher daily tobacco use than those with low symptoms. Receptivity to any tobacco advertising at baseline significantly increased the odds of progression to current use (24.0% vs. 16.9%, AOR = 1.46, 95%CI: 1.29,1.66) and to daily tobacco use (10.3% vs. 6.8%, AOR = 1.41, 95%CI: 1.16,1.72). Compared to the 15–17-year cohort, the 12–14-year cohort had significantly higher rates of progression to both current tobacco use (21.3% vs. 15.4%, AOR = 1.22, 95%CI: 1.07,1.40) and daily use (9.4% vs. 6.5%; AOR = 1.41, 95%CI: 1.17,1.70). There was no difference in progression to ever smoking by age cohort (51.9% vs. 53.9%, AOR = 0.94, 95%CI: 0.85,1.05) (eTable 5).

**Table 1:** Logistic regression of progression to current and daily tobacco use

Baseline (2013-14) Covariates	Response Categories	Sample Size	Tobacco Use at Median 7-years of Follow-up: (range 52 to 98 months)							
			Current Tobacco Use^a^				Daily Tobacco Use^b^			
			%	AOR	95% CI		%	AOR	95% CI	
Age	12–14 yrs	4371	21.3%	1.22	1.07	1.40	9.4%	1.41	1.17	1.70
	15–17 yrs	3125	18.4%	reference			7.0%	reference		
Sex	Male	3728	23.2%	reference			9.6%	reference		
	Female	3768	17.1%	0.63	0.55	0.72	7.1%	0.64	0.54	0.76
Race-ethnicity	NH White	3356	24.8%	reference			11.9%	reference		
	Hispanic	2221	14.3%	0.54	0.45	0.64	3.7%	0.31	0.22	0.42
	NH Black	1096	14.5%	0.47	0.39	0.56	3.8%	0.26	0.18	0.38
	Other	823	18.2%	0.74	0.58	0.95	7.7%	0.68	0.47	0.98
Parental Education	< High School	1567	16.4%	reference			5.9%	reference		
	High School	2878	22.9%	1.25	1.04	1.50	10.2%	1.28	0.99	1.67
	College Grad	3051	19.2%	1.02	0.86	1.21	7.8%	0.99	0.76	1.28
Household Tobacco User	Yes	2183	28.3%	reference			14.1%	reference		
	No	5313	16.9%	0.61	0.53	0.71	6.1%	0.48	0.39	0.58
Smokefree Home	Yes	5820	17.8%	reference			6.9%	reference		
	No	1676	28.1%	1.27	1.07	1.51	13.4%	1.24	1.00	1.54
Internalizing Mental Health Symptoms	Low	5005	18.7%	reference			7.1%	reference		
	Moderate	1849	22.0%	1.08	0.92	1.28	10.8%	1.38	1.10	1.71
	High	642	25.7%	1.27	0.99	1.61	11.2%	1.36	1.04	1.77
Externalizing Mental Health Symptoms	Low	5451	17.8%	reference			7.0%	reference		
	Moderate	1888	25.7%	1.42	1.23	1.65	11.5%	1.43	1.16	1.77
	High	157	30.9%	1.66	1.08	2.55	16.4%	1.98	1.16	3.38
Receptivity to Tobacco Advertising	No	4054	16.9%	reference			6.8%	reference		
	Yes	3442	24.0%	1.46	1.29	1.66	10.3%	1.41	1.16	1.72

^a^Current use at follow-up is defined as using a product for at least 1 day in the past 30 days (adolescents) or reporting currently using a product some days or every day (adults)

^b^Daily use at follow-up is defined as having used a product on more than 25 of the past 30 days (adolescents) or reporting currently using a product every day (adults)

### Product-specific and cross-over effects of receptivity and progression to current and daily product use

Within each tobacco product, we modeled progression to both current use and daily use of the same product by whether adolescents were receptive to the product’s advertising, or receptive to only another tobacco product’s advertising. For current use at follow-up, 6.3% progressed to cigarette smoking, 17.7% to e-cigarette vaping, 3% to cigar smoking and 1.6% to smokeless use (eTable 4). For each product, receptivity to its advertising was associated with progression to current use of that product at follow-up. Receptivity to a different product (but not the target product) was associated with current any tobacco use (AOR = 1.46, 95%CI: 1.29,1.66) as well as use of e-cigarettes (AOR = 1.29, 95%CI: 1.05,1.59), cigars (AOR = 1.40, 95%CI: 1.03,1.88), and smokeless products (AOR = 1.76, 95%CI: 1.02,3.04), but was not statistically significant for cigarettes (AOR = 1.25, 95%CI: 0.96,1.62) (Table 2).

**Table 2:** Models of progression to current or daily use of tobacco product at follow-up survey by receptivity to product-specific advertising or other tobacco product advertising

Product Specific Statistical Model	Product-specific or Other Tobacco Product Receptivity	*N*	Current product use at follow-up [median follow-up = 84 months (range 52–98]						
			Current use^a^				Daily use^b^		
			AOR		95% CI		AOR	95% CI	
Cigarette Model*	no receptivity to any product	4054	reference				reference		
	receptive to other products but not cigarette ads	1687	1.25	0.96		1.62	0.70	0.38	1.29
	receptivity to cigarette ads	1755	1.42	1.07		1.89	1.22	0.70	2.12
E-cigarette model*	no receptivity to any product	4054	reference				reference		
	receptive to other product, but not e-cigarette ads	1071	1.29	1.05		1.59	1.55	1.16	2.06
	receptivity to e-cigarette ads	2371	1.52	1.30		1.77	1.48	1.19	1.84
Cigar Model*	no receptivity to any product	4054	reference				reference		
	receptive to other product, but not cigar ads	2643	1.40	1.03		1.88	0.74	0.16	3.32
	receptivity to cigar ads	799	1.67	1.09		2.56	0.98	0.17	5.70
Smokeless Model*	no receptivity to any product	4054	reference				reference		
	receptive to other product, but not smokeless ads	2083	1.76	1.02		3.04	3.04	0.76	12.09
	receptivity to smokeless ads	1359	2.13	1.20		3.75	3.14	0.95	10.43
Any Product Model*	No receptivity to any product	4054	reference				reference		
	Receptivity to any product	3442	1.46	1.29		1.66	1.41	1.16	1.72

*Each model adjusted for age, sex, race-ethnicity, parental education, household tobacco user, smokefree home,Internalizing and externalizing mental health symptoms

^a^Current use at follow-up is defined as using a product for at least 1 day in the past 30 days (adolescents) or reporting currently using a product some days or every day (adults)

^b^Daily use at follow-up is defined as having used a product on more than 25 of the past 30 days (adolescents) or reporting currently using a product every day (adults)

Progression to daily use by follow-up differed by tobacco product: cigarettes: 12–14 = 1.4%, 15–17 = 1.7%; e-cigarettes: 12–14 = 7.7%, 15–17 = 4.8%; cigars: 12–14 = 0.2%, 15–17 = 0.2%; smokeless: 12–14 = 0.4%, 15–17 = 0.4%. Receptivity to any product-specific advertising was associated with progression to both daily tobacco use (AOR = 1.41, 95%CI: 1.16,1.72). Receptivity to product-specific advertising was associated with progression to daily e-cigarette vaping (AOR = 1.48, 95%CI: 1.19,1.84) but not to daily use of cigarettes, cigars or smokeless tobacco. Evidence for a cross-over effect only occurred for daily e-cigarette vaping where receptivity to other tobacco products, but not e-cigarettes, was associated with progression to daily vaping (AOR = 1.55, 95%CI: 1.16,2.06).

### E-cigarette devices, brands and major flavors used by current vapers at follow-up

At follow-up, 26.4% used cartridge devices (almost half used JUUL, which was the most popular e-cigarette brand); 37.6% used a disposable device (Hyde and Puff Bar were most popular, but still only a third of all types, another third indicated no usual brand); and 27.9% used refillable tank devices (Table 3). Among e-cigarette cartridge vapers, the majority who used JUUL or Vuse vaped menthol, although 29% (95%CI: 20.4%,37.7%) of JUUL users indicated that they had also vaped fruit/candy flavors recently, as did the majority who used other brands or no usual cartridge brand. Only a small percentage of cartridge users vaped a tobacco flavor. Those whose usual device was either disposable or a refillable tank mainly vaped a fruit/candy flavor. While up to one third also recently vaped a menthol flavor, vaping a tobacco flavor was uncommon.

**Table 3:** Characteristics of e-cigarette used on follow-up survey: device type, brand and recent flavorings

Usual Device Type and Brand at Follow-up [median follow-up 84 months, range 52–98]				Any E-cigarette Flavor used in past 30 days								
				Menthol			Fruit/Candy			Tobacco		
Device type	Brand	*n*	%	%	95% CI		%	95% CI		%	95% CI	
Cartridge	JUUL	124	12.1%	78.1	68.6	87.5	29.1	20.4	37.7	8.1	3.4	12.9
	Vuse	27	2.6%	98.6	95.7	100.0	1.4	0	4.3	11.9	0	29.6
	other	32	3.1%	37.3	16.3	58.3	68.6	52.3	84.8	11.4	0	22.9
	No usual	87	8.5%	53.4	42	64.7	58.9	47.6	70.1	4.1	0	8.9
Sub-total			26.4%									
Disposable	Hyde	62	6.1%	24.5	10.4	38.7	92.7	86	99.3	5.6	0	11.7
	Puff Bar	64	6.3%	34.6	21.6	47.6	79.4	67.5	91.3	0		
	Other	135	13.2%	34	25.4	42.5	78.5	70.3	86.8	1.4	0	4.2
	No Usual	124	12.1%	24.1	16.1	32.1	79.2	72	86.5	2	0	4.2
Sub-total			37.6%									
Mod System	various	77	7.5%	21.9	10.1	33.7	86.3	77.6	95	0		
Refillable Tank	various	285	27.9%	23.9	18.7	29.1	87.2	82.8	91.6	1.6	0	3.2
Other		6	0.6%	0	0	0	71.8	68.4	75.2	14	0	44.8
Overall		1023	100%	37.9	34.2	41.5	71.8	68.4	75.2	3.4	2.2	4.7

## Discussion

Over an average of 7 years, half of 2013-14 US adolescent never-tobacco-users progressed to at least try a tobacco product, and at follow-up there were more current as well as daily tobacco users among those who were 12–14 years old at baseline compared to those who were 15–17 years old. Progression was associated with the following expected variables: age, sex, race-ethnicity, both internalizing and externalizing mental health symptoms as well as baseline exposure to a tobacco user in the household and the existence of a smokefree home. Controlled for these variables, receptivity to any tobacco advertising at baseline increased the odds by over 40% that never-tobacco-using adolescents would progress to current or daily tobacco use. Not only was receptivity to product specific advertising associated with current use of the product at follow-up, but receptivity to advertising for a different product was associated with current use of each product except cigarettes. However, for daily use, receptivity to any product advertising was only associated with e-cigarette vaping.

Cross-over effects between receptivity to advertising for one tobacco product brand and initiation with a different product brand has been observed previously with cigarette brands. In the early 1990 s in California, many never-tobacco-users were receptive to the “Joe Camel’ advertising campaign although when they progressed towards smoking, it was most often with Marlboro cigarettes [65]. Also, in the PATH Study, among Wave 1 young-adult never-smokers, exposure to Camel advertising, but not Marlboro or Newport, was associated with smoking initiation with any brand of cigarettes at Wave 2 [44]. However, the image of cigarette smoking has declined considerably over the past two decades [66] and this paper demonstrates that the cross-over effect is no longer restricted to different cigarette brands, but rather to a different nicotine delivery system – e-cigarettes which has become the dominant product category among young people [49, 67]. While there is a cross-over effect from receptivity to cigarette smoking with daily e-cigarette use, receptivity is still associated with current use of cigarettes at follow-up.

Elsewhere, we have confirmed that receptivity to marketing leads to changes in friendship groups so that they include smokers who could provide access to try cigarettes [35]. In this study, at baseline in 2013-14, cigarettes were still the dominant nicotine product used by adolescents, although receptivity among adolescents was already higher for e-cigarettes [20]. Indeed, previously we reported suggestive evidence that there was a cross-over effect in the opposite direction – with receptivity to e-cigarette advertising but not cigarette advertising associated with ever cigarette use in 2015 [42]. But this was before the introduction of the 4th generation of e-cigarettes with their high nicotine concentrations and fruit/candy flavors that masked the harshness of nicotine [52]. These new generations of e-cigarettes quickly surpassed cigarettes as the dominant nicotine product for adolescents [49, 67]. In this longitudinal study, over 80% of never-tobacco-users in our 12–14 year old cohort who progressed to daily use did so with e-cigarettes.

Compared to the 15–17 age cohort, the 12–14 age cohort were much more likely to progress to current and daily use at follow-up. This younger cohort reached mid-adolescence (aged 15–17) years during the huge surge in JUUL sales in 2017 and were much more likely to use JUUL than older cohorts, suggesting increased vulnerability coinciding with the availability of high-nicotine, flavored products [68]. In 2018, under FDA pressure, JUUL discontinued its fruit/candy flavors [69]. Although JUUL lost its market dominance by 2021, it was still the most popular e-cigarette brand in this study, perhaps a testament to the importance of establishing early brand loyalty [70]. It is notable that even though JUUL no longer sells fruit/candy flavored pods, just under one third of vapers who cited JUUL as their usual device noted that they had vaped fruit/candy flavored products recently. This can be done by purchasing “flavor enhancers” to add multiple flavors to JUUL devices, which circumvent FDA regulations [71]. 

This paper adds to the evidence base that e-cigarette marketing has successfully encouraged a new generation to become addicted to nicotine – after a decade in which nicotine addiction had been declining rapidly in this generation. As a category, e-cigarettes have been readily available and marketed in the US since at least 2010 [72] but the FDA was not able to regulate them until 2016 [50]. Since 2019, e-cigarette manufacturers need to apply to the FDA for authorization to market their e-cigarettes. Authorization requires that the product be deemed appropriate for the protection of public health, which is determined by taking into account whether the product increased the likelihood of tobacco cessation among current users, as well as whether its marketing increased the likelihood that non-users would become addicted to nicotine [73]. Post-marketing surveillance in quality US population-based studies has not reported an association between e-cigarettes and successful smoking cessation [74–77]. Our findings highlight how e-cigarette marketing has driven a resurgence of nicotine addiction among adolescents, reversing prior declines in youth tobacco use. These findings also emphasize the importance of increased public health action in both the community (such as advocating for and enforcing flavor bans [78] as a whole as well as in schools suggest the need for robust anti-tobacco media campaigns and more stringent marketing restrictions to protect youth, including interventions and programs that address multiple outcomes (e.g., susceptibility, harm perceptions, countering industry marketing, refusal skills) from varying levels of influence (e.g., policy, media, school, home) that may prevent and reduce vaping among youth [79–83]. 

### Strengths and limitations

A strength of this study was the analysis of a large, nationally representative sample that followed three quarters of never-tobacco-users at W1 for a median of 7 years to assess progression to tobacco use. An additional strength was its measurement of receptivity to a random sample of tobacco advertising images that had recently been used in the US. A limitation is that the study did not include examples of either online or social media marketing. Another limitation was that receptivity to tobacco advertising was only measured at W1 and thus we were not able to assess how receptivity changed over the additional 5–6 surveys that participants completed. We limited our consideration to any and daily use of individual products and did not consider dual use of combustible and non-combustible products. While separating dual use out as an outcome would provide additional insight into patterns of use, such an addition would add considerable complexity which is beyond the scope of this paper,

## Conclusion

This study adds to the evidence that tobacco advertising is associated with adolescent initiation of tobacco use, by demonstrating that over 7 years there is significant progression of never-tobacco-users to both current and daily use. The cross-over advertising effect goes beyond the particular advertised product and effectively promotes daily nicotine use, particularly among 12–14-year-old adolescents, among whom progression was mainly focused on e-cigarettes. The predominance of e-cigarettes, particularly JUULs among new users, illustrates the rapid evolution of tobacco product markets and associated challenges for regulatory bodies. The prominence of flavored products use indicates ongoing appeal despite regulatory efforts limiting such products. Given the continuing shifts in popular brands and device types, ongoing surveillance and adaptive regulatory approaches remain critical. Because advertising receptivity is so strongly associated with initiation of nicotine use, which is a clear public health harm, this calls into question whether there is any benefit that could come from allowing e-cigarette marketing. Comprehensive advertising restrictions and counter-marketing efforts are needed to curb youth tobacco use.

## Supplementary Information

Supplementary Material 1

## Data Availability

All data from the Population Assessment of Tobacco and Health (PATH) Study are publicly available at https://www.icpsr.umich.edu/web/NAHDAP/series/606 (https:/doi.org/10.3886/Series606) and https:/www.icpsr.umich.edu/web/NAHDAP/studies/36231/versions/V38 (https:/doi.org/10.3886/ICPSR36231.v38).
