# Supplementary material for: Tobacco advertising, cross-over effects, and US adolescent progression from never to current tobacco use
Source: BMC Public Health. 2026 Jan 19;26:580. doi: 10.1186/s12889-025-26064-y (PMC12895985; doi:10.1186/s12889-025-26064-y)
Supplement: Supplementary file 1 — Supplementary Material 1 [file 12889_2025_26064_MOESM1_ESM.docx]

**Supplement**

**eTable 1** W1 adolescent participants completion of either W5 or W6 follow-up by characteristics of baseline sample

**eTable 2:** Details of measurement of covariates used in analyses

**eTable 3:** Characteristics of sample who were receptivity to advertising from 4 tobacco products at baseline (W1)

**eTable 4:** Progression to tobacco use at median 7 years follow-up by teen never tobacco

users at baseline by frequency of product used and age

**eTable 5:** Logistic regression of progression of Never Tobacco users at Baseline to ever tobacco use at median 7 years follow-up

**eTable 1** W1 adolescent participants completion of either W5 or W6 follow-up by characteristics of baseline sample


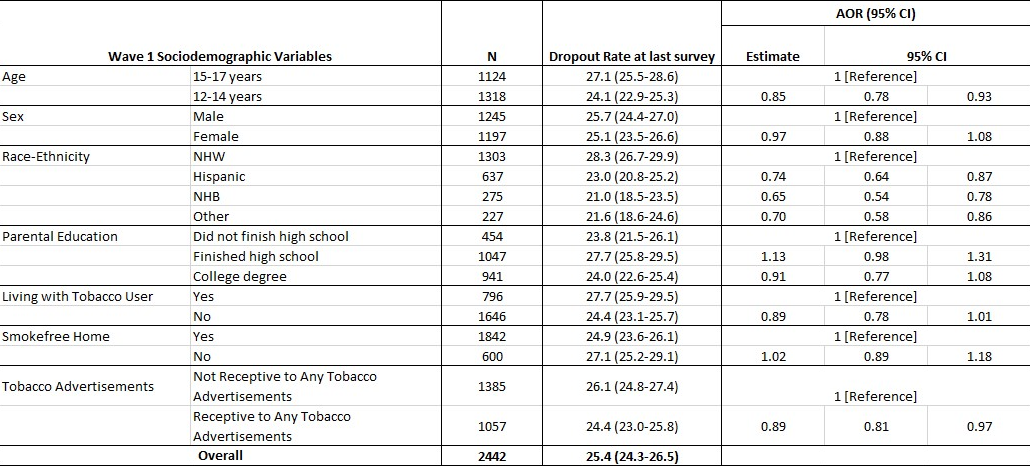


**eTable 2:** Details of measurement of covariates used in analyses

| Sex | Male, female |
| --- | --- |
| Race-Ethnicity | Hispanic, Non-Hispanic White, Non-Hispanic Black and Other. While the Path Study asks participants about a wide range of race-ethnicities, we grouped all but the initial 3 into “other” because of sample size issues. |
| Parental Education | All adolescent interviews included a parental interview which included informed consent. Parental education status was grouped into Less than High School, High School Graduate, College Graduate. |
| Household Tobacco User | Participants reported whether other persons living with them currently used: 1) cigarettes, cigars, cigarillos, filtered cigars 2) smokeless or other tobacco only, 3) no one living in the home uses tobacco. We categorized 1 and 2 as any household tobacco user |
| Smokefree home | Participants were asked which statement best describes rules about smoking a tobacco product that are burned (cigarettes, cigars, pipes or hookah) inside their home. Responses were: 1) not allowed anywhere or anytime; 2)allowed in some places or sometimes; 3) allowed anywhere or anytime. Only the first response was categorized as a smokefree home |
| Internalizing Mental Health Symptoms | The items come from the GAIN-SS*. Participants were asked when they last had significant problems with: 1) feeling very trapped, lonely, sad, blue, depressed or hopeless about the future; 2) sleep trouble such as bad dreams, sleeping restlessly or falling asleep during the day; 3) feeling very anxious, nervous, tense, scared, panicked, or as if something bad was going to happen; 4) becoming very distressed and upset when something reminded you of the past. Responses to all the above items were: past month, 2-12 months age, > 1 year ago, never. |
| Externalizing Mental Health Symptoms | The items come from the GAIN-SS*. Participants were asked when they last did any of the following things at least two times: 1) lied or conned to get things you wanted or to avoid having to do something; 2) had a hard time paying attention at school, work or home; 3) had a hard time listening to instructions at school, work or home; 4) were a bully or threatened other people; started physical fights with other people; felt restless or the need to run around or climb on things; 5) gave answers before the other person finished asking the question. Response categories were: past month, 2-12 months ago, > 1 year ago, never. |

*Global Appraisal of Individual Needs Shore Screener

**eTable 3**: Characteristics of sample who were receptivity to advertising from 4 tobacco products at baseline (W1)

| **Wave 1 Sociodemographic Characteristics and Tobacco Use Predictors** | | **Receptivity to Advertising for Different Tobacco Products** | | | | | | | | | | | | | | | |
| --- | --- | --- | --- | --- | --- | --- | --- | --- | --- | --- | --- | --- | --- | --- | --- | --- | --- |
|  |  | **Sample** | **Cigarette Ads** | | | **E-cigarette Ads** | | | **Cigar Ads** | | | **Smokeless Ads** | | | **Any tobacco ads** | | |
| **Variables** | **Category** | **(N)** | **%** | **95% CI** | | **%** | **95% CI** | | **%** | **95% CI** | | **%** | **95% CI** | | **%** | **95% CI** | |
| **Overall Sample** |  | **7506** | **22.7** | 21.6 | 23.8 | **31.0** | 29.8 | 32.2 | **10.3** | 9.6 | 11.0 | **18.2** | 17.1 | 19.3 | **45.3** | 43.9 | 46.7 |
| Age (years) | 12-14 | 4378 | **21.7** | 20.2 | 23.2 | **29.8** | 28.2 | 31.4 | **9.2** | 8.3 | 10.1 | **16.6** | 15.2 | 17.9 | **43.2** | 41.4 | 44.9 |
|  | 15-17 | 3128 | **24.0** | 22.5 | 25.5 | **32.7** | 30.7. | 34.6 | **11.7** | 10.6 | 12.7 | **20.4** | 18.7 | 22.0 | **48.2** | 46.2 | 50.1 |
| Sex | Male | 3732 | **18.9** | 17.4 | 20.4 | **29.8** | 27.9 | 31.8 | **9.0** | 8.0 | 10.1 | **16.8** | 15.3 | 18.4 | **42.2** | 40.0 | 44.3 |
|  | Female | 3774 | **26.4** | 25.1 | 27.7 | **32.1** | 30.6 | 34.6 | **11.5** | 10.4 | 12.5 | **19.5** | 18.1 | 20.9 | **48.4** | 46.5 | 50.2 |
| Race-Ethnicity | NH White | 3360 | **21.5** | 19.8 | 23.1 | **30.9** | 29.2 | 32.7 | **8.0** | 6.9 | 9.1 | **21.9** | 20.1 | 23.8 | **46.1** | 43.9 | 48.3 |
|  | Hispanic | 2225 | **22.3** | 21.4 | 25.1 | **32.4** | 30.3 | 34.6 | **11.3** | 9.9 | 12.7 | **14.1** | 12.5 | 15.7 | **44.9** | 42.8 | 47.0 |
|  | NH Black | 1097 | **29.2** | 26.5 | 32.0 | **34.3** | 31.4 | 37.1 | **18.4** | 16.2 | 20.7 | **15.5** | 13.4 | 17.5 | **49.7** | 46.4 | 53.0 |
|  | Other | 824 | **18.7** | 15.8 | 21.5 | **24.5** | 21.4 | 27.6 | **7.9** | 6.0 | 9.7 | **13.3** | 10.8 | 15.8 | **36.8** | 33.3 | 40.3 |
| Parental | < HS | 1570 | **23.6** | 21.6 | 25.7 | **30.6** | 28.4 | 32.8 | **13.4** | 11.8 | 15.0 | **15.8** | 14.0 | 17.7 | **44.1** | 41.7 | 46.5 |
| Education | HS | 2881 | **25.2** | 23.3 | 27.1 | **32.9** | 31.1 | 34.7 | **11.5** | 10.2 | 12.9 | **19.5** | 17.8 | 21.2 | **47.7** | 45.5 | 49.9 |
|  | College Grad | 3055 | **20.2** | 18.9 | 21.6 | **29.6** | 27.5 | 31.7 | **8.0** | 7.0 | 9.0 | **18.0** | 16.4 | 19.6 | **43.8** | 41.7 | 45.8 |
| Household | Yes | 2184 | **27.2** | 25.1 | 29.3 | **35.9** | 33.8 | 38.0 | **13.9** | 12.4 | 15.4 | **22.9** | 20.7 | 25.2 | **51.6** | 49.2 | 54.1 |
| Tobacco User | No | 5322 | **20.9** | 19.6 | 22.3 | **29.1** | 27.7 | 30.5 | **8.9** | 8.1 | 9.6 | **16.3** | 15.1 | 17.6 | **42.8** | 41.2 | 44.4 |
| Smokefree Home | No | 1676 | **28.3** | 25.6 | 31.0 | **37.3** | 34.9 | 39.6 | **15.5** | 13.6 | 17.3 | **25.2** | 22.6 | 27.8 | **53.3** | 50.6 | 55.9 |
|  | Yes | 5830 | **21.1** | 19.9 | 22.3 | **29.2** | 27.9 | 30.5 | **8.8** | 8.0 | 9.5 | **16.2** | 15.1 | 17.6 | **43.0** | 41.6 | 44.5 |
| Internalizing Mental Health Symptoms | Low | 5005 | **18.6** | 17.4 | 19.8 | **27.7** | 26.3 | 29.1 | **8.3** | 7.5 | 9.1 | **15.9** | 14.7 | 17.1 | **40.7** | 39.0 | 42.4 |
|  | Moderate | 1849 | **30.2** | 27.9 | 32.5 | **36.1** | 33.8 | 38.3 | **13.7** | 12.2 | 15.2 | **22.5** | 20.2 | 24.8 | **53.6** | 51.1 | 56.2 |
|  | High | 642 | **32.9** | 29.4 | 32.5 | **42.3** | 37.8 | 46.8 | **15.8** | 13.0 | 18.7 | **23.5** | 19.9 | 27.1 | **56.8** | 52.7 | 60.9 |
| Externalizing Mental Health Symptoms | Low | 5451 | **19.8** | 18.6 | 21.0 | **28.1** | 26.7 | 29.5 | **9.1** | 8.4 | 9.8 | **16.3** | 15.1 | 17.5 | **41.5** | 39.9 | 43.1 |
|  | Moderate | 1888 | **29.5** | 27.3 | 31.7 | **38.6** | 36.1 | 41.1 | **13.0** | 11.3 | 14.7 | **22.7** | 20.4 | 25.0 | **55.3** | 52.6 | 58.0 |
|  | High | 157 | **39.2** | 31.3 | 47.0 | **37.9** | 29.2 | 46.7 | **16.6** | 9.9 | 23.4 | **27.7** | 20.4 | 35.0 | **54.6** | 46.6 | 62.6 |

**eTable 4:** Progression to tobacco use at median 7 years follow-up by teen never tobacco

users at baseline by frequency of product used and age

| **Tobacco Product** | **Use at last follow-up** | **12-14 years at baseline** | | | **15-17 years at baseline** | | |
| --- | --- | --- | --- | --- | --- | --- | --- |
|  |  | **estimate** | **95% CI** | | **estimate** | **95% CI** | |
| Cigarette Smoking | Ever use | **24.4** | 23.0 | 25.9 | **29.0** | 27.3 | 30.8 |
|  | Current | **6.3** | 5.5 | 7.0 | **6.3** | 5.6 | 7.1 |
|  | Daily | **1.4** | 1.0 | 1.8 | **1.7** | 1.2 | 2.2 |
| E-cigarette vaping | Ever use | **46.2** | 44.5 | 48.0 | **45.5** | 43.0 | 47.9 |
|  | Current | **17.7** | 16.5 | 18.9 | **12.2** | 10.9 | 13.6 |
|  | Daily | **7.7** | 6.8 | 8.6 | **4.8** | 4.0 | 5.7 |
| Cigar smoking | Ever use | **19.1** | 17.7 | 20.5 | **28.2** | 26.2 | 30.2 |
|  | Current | **3.0** | 2.5 | 3.6 | **4.4** | 3.6 | 5.2 |
|  | Daily | **0.2** | 0.0 | 0.3 | **0.2** | 0.1 | 0.4 |
| Smokeless use | Ever use | **8.7** | 7.7 | 9.6 | **7.6** | 6.6 | 8.6 |
|  | Current | **1.6** | 1.1 | 2.0 | **1.4** | 1.0 | 1.7 |
|  | Daily | **0.4** | 0.1 | 0.6 | **0.4** | 0.2 | 0.7 |
| Any Tobacco Use | Ever use | **51.9** | 50.1 | 53.6 | **53.9** | 51.7 | 56.2 |
|  | Current | **21.3** | 20.0 | 22.7 | **18.4** | 16.9 | 20.0 |
|  | Daily | **9.4** | 8.3 | 10.4 | **7.0** | 6.0 | 7.9 |

**eTable 5**: Logistic regression of progression of never tobacco users at Baseline to ever tobacco use at median 7 years follow-up

| **Covariates** | **Response Categories** | **Ever Tobacco Use at Follow-up** | | | |
| --- | --- | --- | --- | --- | --- |
|  |  | **%** | **AOR** | **95% CI** | |
| Age | 12-14 yrs | 51.9 | 0.94 | 0.85 | 1.05 |
|  | 15-17 yrs | 53.9 | reference | | |
| Sex | male | 53.7 | reference | | |
|  | female | 51.8 | **0.85** | 0.77 | 0.95 |
| Race-ethnicity | NH White | 57.6 | reference | | |
|  | Hispanic | 49.7 | **0.81** | 0.71 | 0.93 |
|  | NH Black | 45.8 | **0.59** | 0.50 | 0.69 |
|  | Other | 46.2 | **0.68** | 0.57 | 0.82 |
| Parental Education | <High School | 48.2 | reference | | |
|  | High School | 54.4 | **1.18** | 1.04 | 1.35 |
|  | College Grad | 53.1 | **1.20** | 1.06 | 1.35 |
| Household Tobacco User | Yes | 62.1 | reference | | |
|  | No | 49.1 | **0.65** | 0.58 | 0.74 |
| Smokefree Home | Yes | 50.4 | reference | | |
|  | No | 61.1 | **1.18** | 1.02 | 1.37 |
| Internalizing Mental Health Symptoms | Low | 49.4 | reference | | |
|  | Moderate | 57.2 | **1.16** | 1.01 | 1.33 |
|  | High | 66.1 | **1.54** | 1.28 | 1.85 |
| Externalizing Mental Health Symptoms | Low | 48.8 | reference | | |
|  | Moderate | 62.8 | **1.49** | 1.33 | 1.67 |
|  | High | 65.0 | 1.48 | 0.97 | 2.27 |
| Receptivity to Tob Advertising | No | 59.9 | reference | | |
|  | Yes | 46.8 | **1.55** | 1.4 | 1.73 |
